# Safety and Feasibility of Intravascular Lithotripsy for Treatment of Below-the-Knee Arterial Stenoses

**DOI:** 10.1177/1526602818783989

**Published:** 2018-06-18

**Authors:** Marianne Brodmann, Andrew Holden, Thomas Zeller

**Affiliations:** 1 Medical University Graz, Austria; 2 Auckland City Hospital, Auckland, New Zealand; 3 Universitäts-Herzzentrum Freiberg–Bad Krozingen, Bad Krozingen, Germany

**Keywords:** arterial stenosis, below the knee, calcification, critical limb ischemia, lithotripsy, calcification, lithoplasty

## Abstract

**Purpose:** To evaluate the safety and feasibility of treating calcified infrapopliteal stenoses using an intravascular lithotripsy (IVL) system. **Methods:** The Disrupt BTK study was a prospective, nonrandomized, multicenter, feasibility, and safety trial that enrolled 20 patients (mean age 79.0±9.6 years; 14 men) at 3 participating sites (*ClinicalTrials.gov* identifier NCT02911623). Fifteen patients had Rutherford category 5 ischemia, and all patients had moderate to severe below-the-knee arterial calcification. Patients were treated with the Shockwave Medical Peripheral IVL System and followed for 30 days. The primary safety endpoint was a composite of major adverse events through 30 days defined as death, myocardial infarction, need for emergency surgical revascularization of the target limb, or amputation of the target limb. The primary effectiveness outcome was acute reduction in the percent diameter stenosis. **Results:** IVL catheter delivery was successful in 19 patients. The composite of major adverse events at 30 days was 0%. The acute reduction in percent diameter stenosis of target lesions was 46.5%. All patients achieved residual diameter stenosis ≤50%. Vascular complications were minimal with only 1 type B dissection reported and 2 stents placed. None of the subjects experienced thrombus formation, abrupt closure, distal embolization, or perforation. There were no device-related complications. **Conclusion:** The early results of this pilot study demonstrated that calcified, stenotic infrapopliteal arteries can be safely and successfully treated with intravascular lithotripsy.

## Introduction

Critical limb ischemia (CLI) is a life-threatening condition associated with significant morbidity and mortality. Within the first year of CLI diagnosis, 25% of patients die and 30% will have a major limb amputation.^1^ Vascular calcification is present in many CLI patients, particularly in the elderly, diabetics, and dialysis-dependent patients. The presence of arterial calcification in CLI patients increases mortality by 50% with a 5-fold increase in the major amputation rate.^2,3^ Arterial calcification may be present in the intima, media, or both. Medial calcification is more prevalent in the infrapopliteal arteries than in the femoropopliteal segment, and nearly 60% of anterior and posterior tibial arteries in CLI patients have medial calcium.^4^ Both intimal and medial calcification contribute to arterial wall stiffness, which leads to the vessel recoil and restenosis seen after endovascular interventions.^2,5–7^ Although considerable progress in endovascular therapy has been made over the past decade, controversy still exists among physicians as to the best endovascular strategy for patients with symptomatic disease of the infrapopliteal arteries, particularly those with significant calcification.

The first use of intravascular lithotripsy (IVL) in femoropopliteal arteries for modification of calcified plaque has been recently described.^8,9^ IVL leverages principles similar to urologic lithotripsy, which has been used as a safe and effective treatment of renal calculi for several decades. Both therapies use pulsatile sonic pressure waves that pass through soft tissue and selectively interact strongly with high-density calcium, producing significant shear stresses that have the ability to fracture the calcium. IVL is designed to safely, effectively, and consistently modify both intimal and medial calcium across a wide range of vascular applications to increase vessel compliance, restore vessel mobility, and provide new versatile treatment options for patients. The goal of this pilot study was to demonstrate that the Shockwave Medical Peripheral IVL System (Shockwave Medical, Fremont, CA, USA) could safely and effectively deliver localized lithotripsy to calcified, stenotic infrapopliteal arteries.

## Methods

### Study Design

The Disrupt Below-the-Knee (BTK) study was a prospective, nonrandomized, multicenter pilot study conducted at 3 sites in Austria, Germany, and New Zealand. The study was designed to demonstrate that the Shockwave Peripheral IVL System could safely and effectively deliver localized pulsatile mechanical energy to calcified infrapopliteal arteries. The inclusion criteria were a >50% infrapopliteal stenosis <150 mm long, a target vessel diameter of 2.5 to 3.5 mm, Rutherford category 1 to 5 ischemia, and moderate to severe calcification. Moderate calcification was defined as densities noted prior to contrast injection in one area of the vessel wall, while severe calcification referred to these densities generally involving both sides of the arterial wall. Computed tomography angiography (CTA) or plain radiography was required to confirm evidence of BTK calcification.

An independent core laboratory (Yale Angiographic Core Laboratory, New Haven, CT, USA) was utilized to provide an unbiased assessment of all imaging utilized in the angiographic outcome assessments. All imaging was performed in accord with the core laboratory’s recommended protocol, which was provided to the sites. Adverse events and reinterventions were documented through a 30-day follow-up.

The ethics committee for each site approved the study protocol and informed consent form, which was signed by all patients prior to study enrolment. The study was conducted in accord with the Declaration of Helsinki, ISO 14155:2011 Guidelines, and Good Clinical Practices. The study was registered on the National Institutes of Health website (*ClinicalTrials.gov*; identifier NCT02911623).

### Study Device

**Figure 1.**
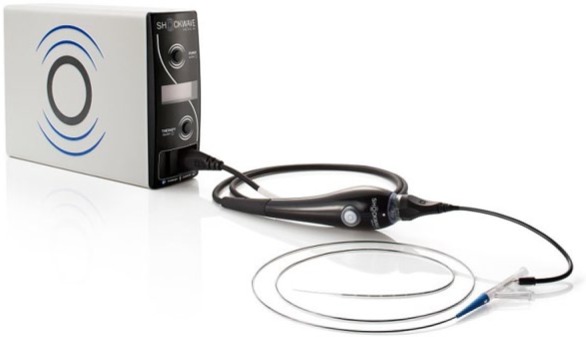
Peripheral intravascular lithotripsy system.

The Shockwave Peripheral IVL System is indicated for lithotripsy-enhanced, low-pressure balloon dilation of calcified, stenotic peripheral arteries in patients who are candidates for percutaneous therapy. The IVL device delivers pulsatile sonic pressure waves locally to effectively modify vascular calcium in a safe manner. The system consists of a generator, a connector cable, and a catheter that houses an array of lithotripsy emitters enclosed in an integrated balloon (Figure 1). Once a calcified arterial lesion is crossed with a 0.014-inch guidewire, the IVL catheter is advanced to the lesion and positioned using radiopaque marker bands. The generator produces 3 kV of energy that travels through the connector cable and catheter to the lithotripsy emitters at 1 pulse/s. With the integrated balloon expanded to 4 atmospheres using a mixture of saline and contrast solution to achieve balloon–vessel wall apposition without significant angioplasty, a small electrical discharge at the emitters vaporizes the fluid and creates a rapidly expanding bubble within the balloon. This bubble generates a series of sonic pressure waves that travel through the fluid-filled balloon and pass through soft vascular tissue, selectively cracking the hardened, calcified plaque. The emitters positioned along the length of the device create a localized field effect within the vessel to fracture both intimal and medial calcium.

The integrated balloon plays a unique role unlike traditional angioplasty balloons; first, its apposition to the vessel wall facilitates efficient energy transfer, and second, it safely constrains the expansion of the bubble. Following calcium disruption, the integrated balloon is inflated to nominal pressure (6 atm) to maximize lumen gain. This cycle is then repeated as needed until the desired diameter is obtained. The IVL catheter can be moved to other lesion locations to deliver lithotripsy. For this study, the 60-mm-long IVL catheter was available in 5 diameters: 2.5, 2.75, 3.0, 3.25, and 3.5 mm.

### Study Procedures

Vascular access, anticoagulation, and introduction of guidewires and catheters were in accord with each institution’s standard of care for endovascular procedures. Baseline angiography with runoff and 2 orthogonal views of the target lesion was required to assess target lesion characteristics and to determine final patient eligibility. Eligible subjects were enrolled in the study if the angiographic inclusion criteria were met and guidewire passage through the target lesion had been achieved. Predilation of the target lesion was at physician discretion to facilitate crossing of this early-generation IVL catheter. After the target lesion was successfully crossed with a 0.014-inch wire, the IVL catheter was advanced to the target lesion and multiple pulses were applied at the treating physician’s discretion until a satisfactory result was obtained. Completion angiography, including runoff and 2 orthogonal views of the treated vessel, was performed to assess the final result.

### Study Outcomes

The primary safety endpoint was a composite of major adverse events (MAE) through 30 days, defined as death, myocardial infarction, need for emergency surgical revascularization of the target limb, or amputation of the target limb. The primary effectiveness outcome was acute reduction in percent diameter stenosis of the target lesion. The secondary study outcome was procedural success, defined as the ability of the Shockwave Peripheral IVL System to achieve ≤50% residual diameter stenosis. Continuous variables are expressed as mean ± standard deviation (range); categorical variables are given as the counts.

### Patient Enrollment

**Figure 2.**
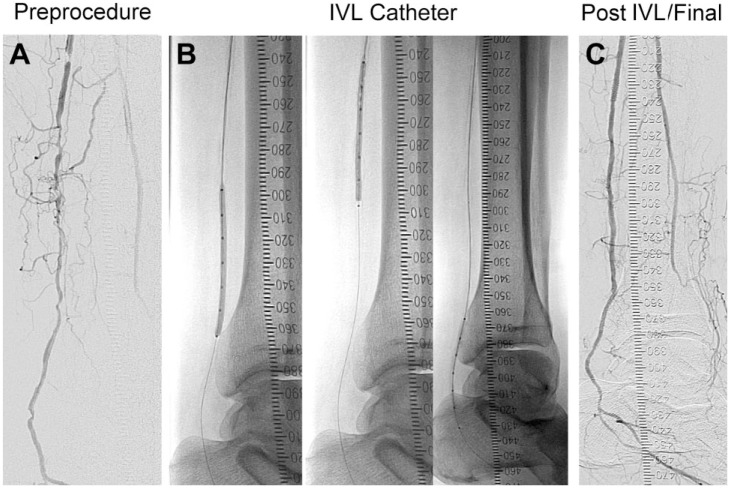
In this illustrative case (A) angiography documented a reference vessel diameter of 2.5 mm, a diameter stenosis of 95.9%, and a lesion length of 144 mm in the posterior tibial artery. (B) A 2.75-mm intravascular lithotripsy (IVL) catheter delivered 120 pulses with no pre- or postdilation. (C) Final angiography recorded a 23.6% residual stenosis and no vascular complications.

Between June 22, 2016, and March 9, 2017, 20 consecutive patients (mean age 79.0±9.6 years; 14 men) meeting all eligibility criteria were enrolled across the 3 sites. Baseline demographics, medical history, and lesion characteristics are shown in Table 1. Patients enrolled in the study were burdened by the comorbidities typical in peripheral artery disease (PAD) patients, including hypertension, hyperlipidemia, diabetes, and renal insufficiency; 16 had CLI (15 Rutherford category 5 and 1 category 4). The mean reference vessel diameter was 3.2 mm and the mean minimum lumen diameter was 0.9 mm, with a corresponding percent diameter stenosis of 72.6%. The mean lesion length was 52.2±35.8 mm. All patients had moderate to severe calcification. Calcium burden was significant with the average length of calcium (91.8 mm) being greater than the lesion length. The majority of subjects (n=17) had 1 target artery, while 2 subjects had 2 target arteries. The anterior and posterior tibial arteries were the most commonly treated vessels (Figure 2).

**Table 1.** Baseline Patient and Angiographic Characteristics of the 20 Study Patients.^a^

Age, y	79±9.6
Men	14
Diabetes	8
Hypertension	19
Hyperlipidemia	15
Renal insufficiency	8
Coronary artery disease	8
Current or former smoker	5
Rutherford category	
3	4
4	1
5	15
Lesion location (n=21)	
Tibioperoneal trunk	2
Anterior tibial	8
Posterior tibial	8
Peroneal	2
BTK popliteal artery	1
RVD, mm	3.2±0.6 (2.4–4.8)
Lesion length, mm	52.2±35.8 (13.8–144.0)
Calcified length, mm	72.1±37.6 (12.4–172.6)
Calcification^b^	
Moderate	11
Severe	9
Lumen diameter, mm	0.9±0.6 (0.0–1.9)
Diameter stenosis, %	72.6

Abbreviations: BTK, below the knee; RVD, reference vessel diameter.

a Continuous data are presented as the means ± standard deviation (range); categorical data are given as the counts.

b Calcification as defined by the core laboratory was readily apparent densities noted within the apparent vascular wall at the site of a stenosis. Classification included none/mild, moderate (densities noted only prior to contrast injection), and severe (radiopacities noted prior to contrast injection generally involving both sides of the arterial wall).

## Results

Access to the target lesion and treatment delivery of the IVL catheter was successful in 19 patients (Table 2). In 1 subject the IVL catheter could not be delivered, and the procedure was aborted without complications. The infrapopliteal procedures were completed with a low use of adjunctive therapies including pre- and postdilation balloons (5 and 3, respectively). Two stents were placed for residual stenosis but none for flow-limiting dissections. The mean total procedure time was 79 minutes (range 30–168). Mean fluoroscopy time was 18.1±14.2 minutes. An average of 1.3 IVL catheters per patient were used for treatment, and an average of 77.8 lithotripsy pulses were delivered. The mean balloon inflation pressure was 6 mm Hg (range 4–9).

**Table 2.** Procedure Characteristics and Angiographic Results.^a^

Procedure	
Duration,^b^ min	79±1.5 (30–168)
Fluoroscopy time, min	18.1±14.2 (5.5–68.0)
Contrast, mL	58±37 (40–178)
Predilation	5/21
Postdilation	3/21
Pressure, mm Hg	6±1.2
Stents	2/21
IVL pulses	77.8±37.0
Successful IVL delivery	19/20
Angiographic outcomes	
Lumen diameter, mm	2.4±0.5 (1.5–3.6)
Diameter stenosis, %	26.2±9.3
Stenosis reduction, %	46.5±18.6
Acute gain, mm	1.5±0.5 (0.7–2.3)
Dissection	1
Perforation	0
Distal embolization	0
Thrombus	0
No reflow	0
Abrupt closure	0

Abbreviation: IVL, intravascular lithotripsy.

a Continuous data are presented as the means ± standard deviation (range); categorical data are given as the counts.

b Defined as the time from the start of access to procedure completion.

All subjects completed the 30-day follow-up. There was no death, myocardial infarction, revascularization, or amputation (MAE 0%). The reduction in percent diameter stenosis was 46.5%. Success (≤50% residual stenosis) was achieved in all 19 procedures in which the Shockwave IVL catheter could be positioned. Final angiographic characteristics are summarized in Table 2. Posttreatment mean diameter stenosis was 26.2%, representing an acute mean lumen gain of 1.5±0.5 mm (range 0.7–2.3). Vascular complications were minimal, with only 1 grade B dissection reported. None of the subjects experienced thrombosis, abrupt closure, distal embolization, or perforation. Five patients had 10 adverse events, including 6 vascular complications, 1 non–target leg angioplasty, 1 minor amputation, 1 wound healing disorder, and 1 contrast allergy. None of the events was adjudicated as related to the study device.

## Discussion

This pilot study demonstrated that localized IVL with the Shockwave Medical catheter can be performed safely and effectively in infrapopliteal lesions. The acute results demonstrated low residual stenosis without relevant vascular complications or any MAEs. These early outcomes in a heavily calcified BTK cohort are consistent with results obtained from the pooled Disrupt PAD I and II studies,^8,9^ which evaluated the use of the Shockwave IVL for femoropopliteal disease. In those studies, 95 patients were treated at 8 centers, achieving a low average residual stenosis of 23.8% and acute gain of 3.0 mm, with only 1 stent implanted for a single grade D dissection (the only MAE). Overall, 76.7% of patients were patent at 6 months in this historically difficult-to-treat population, with a 96.8% freedom from target lesion revascularization.^9^

The BTK device was simple to use, combining the calcium-disrupting capability of lithotripsy with the familiarity of traditional catheter-based interventional devices. The IVL catheter used in this study had a crossing profile of 0.58 to 0.59 inches, and the catheter was able to be delivered to all but 1 lesion. A future iteration of the device will have a crossing profile of 0.52 to 0.54 inches and hydrophilic coating to aid in lesion crossing.

The acute success of traditional endovascular therapies may be hampered by vessel recoil, dissection, or inability to achieve adequate dilation due to the presence of heavy calcification. A recent study assessing CLI patients treated with balloon angioplasty found early recoil in 97% of patients, resulting in a 29% reduction in the minimum lumen diameter that could contribute to the restenosis frequently seen following dilation alone.^10^ Long-term failure of BTK endovascular therapy may occur due to vessel or in-stent restenosis, which has been reported in up to 77% of patients.^11^ BTK restenosis is heavily influenced by the presence of medial calcification. In this pilot study, the Shockwave Peripheral IVL System demonstrated high procedural success and an excellent safety profile and was a stand-alone treatment in the majority of cases.

Current treatment strategies for calcified arteries have resulted in an increased risk for adverse events due to the fact that they do not differentiate between the calcific lesion and soft tissue. IVL is the only technology that addresses both intimal and medial calcium and does so with minimal vessel injury because of its ability to selectively differentiate calcium from soft tissue.

### Limitations

The Disrupt BTK study enrolled a small number of patients with short-term follow-up. Future studies with a larger number of patients and long-term follow-up will be needed to further evaluate this novel technology.

## Conclusion

Early results with the Shockwave Peripheral Intravascular Lithotripsy System in infrapopliteal lesions show a consistent reduction in stenosis and no procedural complications, including perforation or distal embolization. Additional evaluation with this promising technology is warranted.
